# The Ohio Amended Law—Amended

**Published:** 1892-05

**Authors:** 


### EDITORIAL.

### The Ohio Dental Law—Amended.

As will be seen by reference to another page of this number of the REGISTER, the law regulating the practice of Dentistry in Ohio has been amended by the recent Legislature.

By the amendments and changes made, the law is brought up to the present time so that it embodies the leading features of the laws enacted in other States, viz:

The registration of all practitioners in the State, this registration is to be made with the Board of Examiners, and there only.

The examination of all who begin the practice in Ohio since July 4, 1889, except those who hold a diploma from a reputable Dental College or a license under the former law.

A Board of Examiners, consisting of five persons, is provided for, which is to be appointed by the Governor; rules and regulations are given for the performance of the duties of this Board. Provision is made for the prompt and efficient execution of the law, and a penalty that will be efficient in all cases of conviction. False assumption of a name or title, whereby the public may be deceived, is a misdemeanor, subjecting the violator to the penalties prescribed.

The law took effect immediately upon its passage, which was April 7, 1892.

It is to be hoped that every reputable dentist in the State will give the law his hearty support. If any are found ignorantly violating it, it is the duty of those more highly favored to see to it that such persons are promptly informed as to the requirements of the law; and those who knowingly transgress should be made to realize that it is not a good thing to violate a wholesome law. Those who promptly, willingly and cheerfully comply with beneficial law cannot afford to permit others about them to ignore requirements that are for the public and professional well-being.

